# Association Between Dietary Fiber Intake and Non-alcoholic Fatty Liver Disease in Adults

**DOI:** 10.3389/fnut.2020.593735

**Published:** 2020-11-19

**Authors:** Huimin Zhao, Aihua Yang, Lina Mao, Yaning Quan, Jiajia Cui, Yongye Sun

**Affiliations:** ^1^Department of Nutrition and Food Hygiene, School of Public Health, Qingdao University, Qingdao, China; ^2^Dept. of Clinical Laboratory, The Affiliated Hospital of Qingdao University, Qingdao, China; ^3^Qingdao Fuwai Cardiovascular Hospital, Qingdao, China

**Keywords:** Non-alcoholic fatty liver disease (NAFLD), dietary intake, cereal fiber, fruit fiber, vegetable fiber, dose-response, National Health and Nutrition Examination Survey (NHANES)

## Abstract

**Background:** Evidence on the association of non-alcoholic fatty liver disease (NAFLD), a public health concern, with dietary fiber intake is inconsistent.

**Objective:** To investigate the relationship between dietary fiber intake from different sources and NAFLD risk in US adults.

**Methods:** Data were collected from the 2007–2014 National Health and Nutrition Examination Survey. NAFLD was defined as a United States Fatty Liver Index ≥30, and dietary fiber intake was assessed through two 24-h dietary recall interviews. Logistic regression and restricted cubic spline models were used to explore the relationship of dietary intakes of total, cereal, fruit, and vegetable fiber with NAFLD risk.

**Results:** A total of 6,613 participants, aged more than 20 years, were included in this study. After adjusting for multiple confounding factors, the odds ratios and 95% confidence intervals of NAFLD for the highest quartile vs. lowest quartile intakes of total, cereal, fruit, and vegetable fiber were 0.12 (0.08–0.16), 0.25 (0.19–0.33), 0.41 (0.33–0.52), and 0.42 (0.32–0.56), respectively. In stratified analyses by sex and age, statistically significant negative associations of dietary intakes of total, cereal, fruit, and vegetable fiber with NAFLD risk were observed in all participants. Dose-response analysis indicated a non-linear correlation between NAFLD risk and dietary intake of total fiber, whereas the relationship was linear for cereal, fruit, and vegetable fiber intakes.

**Conclusion:** Total, cereal, fruit, and vegetable fiber intakes exhibit negative correlations with NAFLD risk in the general adult population in the United States.

## Introduction

Non-alcoholic fatty liver disease (NAFLD) is a clinicopathological syndrome characterized by excessive fat deposition in hepatocytes in the absence of definite liver-damaging factors, such as alcohol intake (1). NAFLD progresses from intracellular fat accumulation to liver fibrosis, cirrhosis, and, ultimately, liver failure (2–5). Growing evidence indicates that NAFLD is a multisystem disease that increases the risk of cardiovascular disease, type 2 diabetes, and other chronic diseases (6–8). NAFLD is an emerging health problem with high worldwide prevalence (9, 10); the estimated prevalence rate of approximately 30% among American adults (11). Because no effective medical treatment for NAFLD has been reported (12), identifying potential modifiable factors to control or prevent the development of NAFLD is necessary.

Several lifestyle and dietary factors, regarded as modifiable conditions, have been linked to NAFLD. Intakes of fried foods, refined grains, processed meat, and fructose-rich foods have been reported to increase the risk of NAFLD (13–15), whereas intakes of whole grains, legumes, probiotic dairy products, vegetables, and fruits have been shown to decrease the NAFLD risk (15–18). Some studies have confirmed that high intake of dietary fiber, which is found predominantly in cereals, fruits, and vegetables, was associated with decreased risk of type 2 diabetes, hypertension, hyperuricemia, cardiovascular disease, and cancer (19–25). Additionally, the relationship between dietary fiber intake and NAFLD has been reported. A cross-sectional study in the Netherlands demonstrated that dietary fiber intake was low among participants with a high fatty liver index (26). Another large cross-sectional study in China demonstrated the association of total dietary fiber intake with a low prevalence of newly diagnosed NAFLD (27). Furthermore, a case–control study in Iran demonstrated that dietary fiber intake in patients with NAFLD was lower than that in healthy controls (28). However, another cross-sectional study in Israel found no significant difference in dietary fiber intake between NAFLD and non-NAFLD groups (29), and a cross-sectional study in China revealed higher dietary fiber intake among participants with NAFLD than controls (30). Taken together, the results of studies investigating the association between dietary fiber intake and NAFLD are inconsistent.

Some of the above studies did not adjust for any confounders, so they could not reflect the true relationship between dietary fiber intake and NAFLD. Additionally, none of the studies investigated the dose-response relationship between dietary fiber intake and NAFLD or analyzed the dietary fiber intake presented in per kilogram (kg) of body weight or per kilocalorie (kcal) of energy intake. Therefore, we explored the associations of intakes of total fiber and cereal, fruit, and vegetable fiber with NAFLD risk in the general adult population in the United States by using data from the 2007–2014 National Health and Nutrition Examination Survey (NHANES).

## Methods

### Study Population

**Figure 1:**
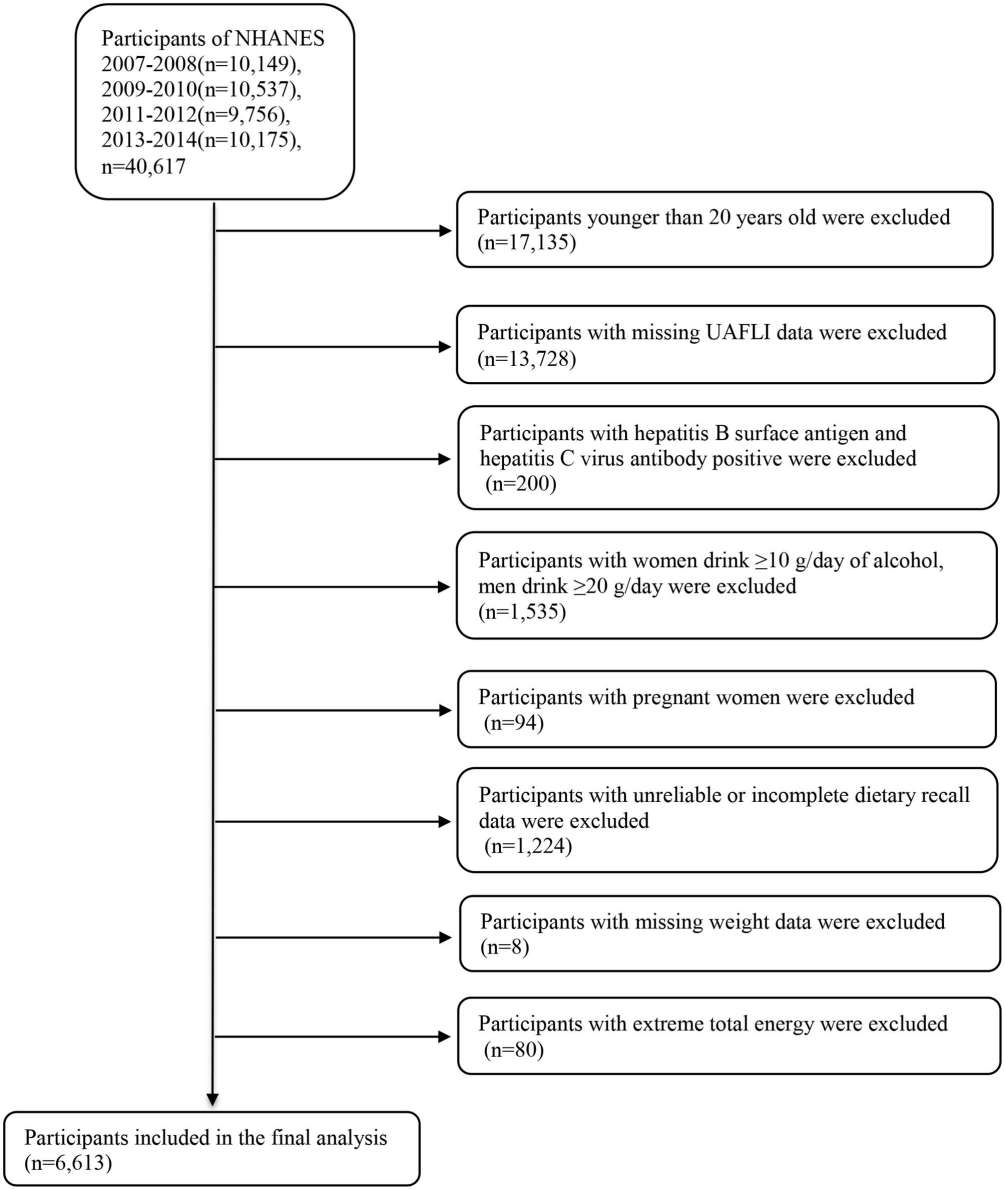
Flow chart of the selection process. NHANES, National Health and Nutrition Examination Survey.

We combined the publicly available data from four NHANES datasets, namely those of 2007–2008, 2009–2010, 2011–2012, and 2013–2014, for analysis (https://wwwn.cdc.gov/nchs/nhanes/Default.aspx). The 2007–2014 NHANES datasets included data on a total of 40,617 participants; however, our analysis was limited to 23,482 participants aged 20 years and older. We excluded participants with missing information to calculate the United States fatty liver index (USFLI; *n* = 13,728). Participants positive for hepatitis B surface antigen and hepatitis C virus antibodies (*n* = 200) were also excluded. Additionally, we excluded individuals with elevated alcohol intake (≥10 g/day for females and ≥20 g/day for males; *n* = 1,535). We also excluded pregnant women (*n* = 94), participants with unreliable or incomplete dietary recall (*n* = 1,224) and missing weight data (*n* = 8), and participants with average energy intake > mean + 3SD (4,261 kcal) or < mean – 3SD (0 kcal) (*n* = 80). Finally, our analysis included 6,613 individuals comprising 3,067 men and 3,546 women (Figure 1). The Review Board of the National Center for Health Statistics granted the approval for using the NHANES data, and all participants provided informed consent.

### NAFLD Measurement

We defined NAFLD on the basis of the USFLI. As described in previous articles, we calculated USFLI on the basis of race, age, gamma glutamyl transferase level, waist circumference, fasting insulin level, and fasting glucose level, and the cutoff value of 30 was used to define NAFLD (31). USFLI has been reported to be a reliable non-invasive NAFLD measurement and an independent predictor of overall and liver-related mortality (32–34).

### Dietary Fiber Intake

Dietary fiber intake was assessed using two 24-h dietary recall interviews. The first interview was conducted at mobile examination center, and the second interview was performed after 3–10 days by telephone. (The specific measuring guides see the website: https://www.cdc.gov/nchs/nhanes/measuring_guides_dri/measuringguides.htm). Nutrient intakes were calculated on the basis of the United States Department of Agriculture Food and Nutrient Database for Dietary Studies (35). Average daily dietary fiber intakes were calculated and adjusted to body weight (22). Intakes of dietary fiber (mg/kg/day) were categorized according to quartiles.

### Study Covariates

Factors included in regression models to control the potential effects of confounding variables were the following: age (20–44 y, 45–59 y, 60–74 y, and ≥75 y), sex (male and female), race (Mexican-Americans, other Hispanics, non-Hispanic Whites, non-Hispanic Blacks, and other races), education level (under high school, high school, and above high school), annual household income (<$20,000, $20,000–$44,999, $45,000–$74,999, and ≥$75,000), smoking status (smoking at least 100 cigarettes in life or not), vigorous recreational activity (yes or no), diabetes (yes or no), hypertension (yes or no), average daily energy intake, levels of serum total cholesterol (TC) and uric acid (UA). Diabetes was defined as a fasting blood glucose level ≥7.0 mmol/L, 2-h plasma glucose level ≥11.1 mmol/L, use of diabetes pills or insulin, or self-reported diabetes diagnosis (36). Hypertension was defined as mean systolic blood pressure ≥130 mmHg, mean diastolic blood pressure ≥80 mmHg, use of prescription drugs for hypertension, or self-reported hypertension diagnosis (37).

### Statistical Analysis

Stata 15.0 was used for all statistical analyses. According to the NHANES analysis guidelines (38), new 8-year weights could be calculated by dividing the 2-year weights by four (the number of 2-year cycles). Main characteristics of participants are presented as mean ± standard deviation or median (interquartile ranges) for continuous variables and as frequencies (percentage) for categorical variables. Differences between participants with and without NAFLD were assessed using Student's *t*-test (for continuous variables with normal distribution) or non-parametric test (for non-normal distribution). Differences in categorical variables were evaluated using chi-square tests. Intakes of dietary fiber were categorized according to quartiles; quartile 1 was used as a reference category. The binary logistic regression model was used to analyze the relationship between NAFLD and intakes of total fiber and cereal, fruit, and vegetable fiber. Model 1 was adjusted for age and sex. Model 2 was further adjusted for race, educational level, smoking status, recreational activities, annual household income, hypertension, diabetes, average daily energy intake, and UA and TC levels. Stratified analysis was conducted by sex (male and female) and age (20–44 y and ≥45 y age groups) to determine the relationship between dietary fiber intake and NAFLD. Odds ratios (ORs) and 95% confidence intervals (CIs) were calculated from logistic regression analyses. Dose-response relationships were evaluated using a restricted cubic spline function with three knots located at the 5, 50, and 95th percentiles of the exposure distribution in the fully adjusted model. The non-linear *p*-value was calculated by testing the value of the quadratic zero spline coefficient. A two-tailed *p*-value <0.05 was considered statistically significant.

## Results

Table 1 presents the comparison of baseline characteristics between NAFLD and non-NAFLD groups according to sex. Of 6,613 subjects, the overall prevalence of NAFLD was 36.70% (41.93% for males and 32.09% for females). Irrespective of sex, the NAFLD group, compared with the non-NAFLD group, tended to be older, Mexican-American, and smokers. Moreover, the NAFLD group exhibited a higher number of participants with hypertension or diabetes, higher body mass index, and higher serum UA level, whereas the education level, income, vigorous recreational physical activity level, and total fiber intake and cereal, fruit, and vegetable fiber intakes were lower in the NAFLD group (all *p* < 0.05).

**Table 1:** Baseline characteristics of the participants by NAFLD, U.S. adult aged ≥20 years, NHANES 2007-2014.

	**NAFLD (total)**			**NAFLD (men)**			**NAFLD (women)**		
**Characteristic**	**No**	**Yes**	***P*-value**	**No**	**Yes**	***P*-value**	**No**	**Yes**	***P-*value**
Number of participants (%)	4189 (63.30%)	2424 (36.70%)		1781 (58.07%)	1286 (41.93%)		2408 (67.91%)	1138 (32.09%)	
Age group (*n*, %)			<0.001			<0.001			<0.001
20–44 years	1890 (73.58%)	709 (26.42%)		805 (68.64%)	388 (31.36%)		1085 (78.08%)	321 (21.92%)	
45–59 years	988 (61.09%)	655 (38.91%)		427 (55.58%)	328 (44.42%)		561 (65.86%)	327 (34.14%)	
60–74 years	862 (54.53%)	742 (45.47%)		346 (46.76%)	389 (53.24%)		516 (60.77%)	353 (39.23%)	
≥75 years	449 (58.35%)	318 (41.65%)		203 (52.77%)	181 (47.23%)		246 (62.57%)	137 (37.43%)	
Race (*n*, %)			<0.001			<0.001			<0.001
Mexican American	488 (49.42%)	552 (50.58%)		204 (43.45%)	281 (56.55%)		284 (55.30%)	271 (44.70%)	
Other Hispanic	444 (64.17%)	294 (35.83%)		175 (61.42%)	144 (38.58%)		269 (66.54%)	150 (33.46%)	
Non-Hispanic White	1845 (63.88%)	1155 (36.12%)		789 (58.02%)	650 (41.98%)		1056 (69.03%)	505 (30.97%)	
Non-Hispanic Black	931 (79.05%)	269 (20.95%)		394 (79.30%)	120 (20.70%)		537 (78.90%)	149 (21.10%)	
Other Race	481 (75.31%)	154 (24.69%)		219 (69.77%)	91 (30.23%)		262 (80.32%)	63 (19.68%)	
Educational Level (*n*, %)			<0.001			0.005			<0.001
< High school	902 (54.75%)	802 (45.25%)		398 (52.22%)	396 (47.78%)		504 (56.95%)	406 (43.05%)	
High school	954 (64.19%)	543 (35.81%)		418 (59.88%)	298 (40.12%)		536 (67.93%)	245 (32.07%)	
>High school	2,329 (68.21%)	1,075 (31.79%)		964 (61.40%)	590 (38.60%)		1,365 (74.03%)	485 (25.97%)	
Smoking status (*n*, %)			<0.001			<0.001			0.010
Yes	1,576 (59.85%)	1,131 (40.15%)		840 (54.49%)	705 (45.51%)		736 (66.21%)	426 (33.79%)	
No	2,611 (68.28%)	1,293 (31.72%)		940 (63.83%)	581 (36.17%)		1,671 (71.36%)	712 (28.64%)	
Vigorous recreational activity (*n*, %)			<0.001			<0.001			<0.001
Yes	1,031 (81.56%)	270 (18.44%)		571 (77.05%)	192 (22.95%)		460 (87.79%)	78 (12.21%)	
No	3,158 (59.87%)	2,154 (40.13%)		1,210 (52.21%)	1,094 (47.79%)		1,948 (65.59%)	1,060 (34.41%)	
Hypertension (*n*, %)			<0.001			<0.001			<0.001
Yes	1,805 (51.22%)	1,639 (48.78%)		810 (47.10%)	865 (52.90%)		995 (55.09%)	774 (44.91%)	
No	2,384 (77.23%)	785 (22.77%)		971 (71.57%)	421 (28.43%)		1,413 (81.74%)	364 (18.26%)	
Diabetes (*n*, %)			<0.001			<0.001			<0.001
Yes	505 (32.45%)	910 (67.55%)		229 (30.26%)	461 (69.74%)		276 (34.43%)	449 (65.57%)	
No	3,684 (71.33%)	1,514 (28.67%)		1,552 (65.40%)	825 (34.60%)		2,132 (76.38%)	689 (23.62%)	
Annual household income (*n*, %)			<0.001			<0.001			<0.001
< $20,000	780 (61.28%)	561 (38.72%)		266 (58.77%)	235 (41.23%)		514 (62.56%)	326 (37.44%)	
$20,000–$44,999	1,352 (58.95%)	931 (41.05%)		549 (51.12%)	486 (48.88%)		803 (64.89%)	445 (35.11%)	
$45,000–$74,999	781 (64.37%)	409 (35.63%)		340 (57.31%)	237 (42.69%)		441 (70.95%)	172 (29.05%)	
≥$75,000	1,098 (72.33%)	423 (27.67%)		535 (66.74%)	277 (33.26%)		563 (78.63%)	146 (21.37%)	
BMI (kg/m^2^)	26.00 (23.20, 29.10)	33.14 (29.60, 37.80)	<0.001	26.00 (23.60, 28.50)	31.88 (28.81, 35.47)	<0.001	26.00 (22.70, 29.60)	35.00 (30.80, 40.23)	<0.001
TC (mg/dL)	189 (165, 215)	192 (166, 220)	0.020	184 (161, 208)	189 (162, 217)	0.002	194 (168, 221)	195 (171, 223)	0.222
UA (mg/dL)	5.0 (4.2, 5.9)	6.0 (5.2, 6.9)	<0.001	5.7 (5.1, 6.4)	6.4 (5.7, 7.3)	<0.001	4.5 (3.9, 5.2)	5.6 (4.8, 6.3)	<0.001
Average energy intake (kcal/day)	1,869.5 (1,463.5, 2,386.5)	1,942.0 (1,479.0, 2,464.0)	0.581	2,225.5 (1,780.0, 2,762.0)	2,176.0 (1,719.0, 2,710.0)	0.064	1,639.0 (1,336.5, 2,036.5)	1,665.5 (1,335.5, 2,112.0)	0.125
Total fiber intake (mg/kg/day)	216.60 (147.70, 310.90)	156.68 (110.19, 219.75)	<0.001	222.80 (151.39, 313.95)	163.93 (114.78, 235.45)	<0.001	213.46 (144.14, 305.60)	149.28 (108.13, 205.14)	<0.001
Cereal fiber intake (mg/kg/day)	90.54 (54.83, 139.35)	68.70 (41.53, 101.16)	<0.001	97.35 (57.21, 153.94)	73.43 (43.86, 109.31)	<0.001	86.15 (53.40, 130.65)	64.60 (39.26, 93.75)	<0.001
Fruit fiber intake (mg/kg/day)	23.14 (1.48, 54.93)	13.34 (0,34.05)	<0.001	19.40 (0, 51.36)	11.45 (0, 31.41)	<0.001	26.60 (2.60, 57.52)	15.04 (0, 37.41)	<0.001
Vegetable fiber intake (mg/kg/day)	38.68 (18.34, 66.09)	27.29 (13.51, 41.95)	<0.001	36.54 (16.53, 62.57)	27.22 (13.39, 48.33)	<0.001	40.47 (19.79, 69.28)	27.49 (13.77, 47.00)	<0.001

*BMI, body mass index; TC, total cholesterol; UA, uric acid. Data are presented as participants (percentage) for categorical variables or 50th (25th, 75th) for continuous variables*.

Table 2 presents the weighted ORs (95% CIs) of NAFLD based on quartiles of total fiber and cereal, fruit, and vegetable fiber intakes. Univariate logistic regression analysis demonstrated the association of intakes of total fiber and cereal, fruit, and vegetable fiber with decreased NAFLD risk. Compared with the lowest quartile, the ORs (95% CI) of NAFLD for the highest quartile intake of total fiber, cereal fiber, fruit fiber, and vegetable fiber were 0.20 (0.16–0.25), 0.32 (0.26–0.40), 0.44 (0.36–0.53), and 0.41 (0.33–0.51), respectively. After adjusting for age and sex (model 1), the results were similar to the crude ORs (95% CIs). After further adjusting for race, education level, smoking status, vigorous recreational activities, hypertension, diabetes, income, average daily energy intake, UA level, and TC level (model 2), dietary fiber (various sources) intakes still exhibited a negative association with NAFLD risk. We further analyzed the associations of NAFLD with dietary fiber intakes as mg/kcal/day, and the inverse association between total fiber intake and NAFLD was still significant in all models (Supplementary Table 1).

**Table 2:** Weighted ORs and 95% CIs for NAFLD according to the quartiles of dietary fiber intake.

	**Crude**	**Model 1**	**Model 2**
	**OR (95%CI)**	**OR (95%CI)**	**OR (95%CI)**
Total fiber intake (mg/kg/day)			
≤ 128.70	1.00 (ref.)	1.00 (ref.)	1.00 (ref.)
>128.70–191.02	0.73 (0.60–0.88)**	0.69 (0.56–0.84)**	0.59 (0.45–0.78)**
>191.02–279.26	0.42 (0.34–0.51)**	0.37 (0.30–0.46)**	0.28 (0.21–0.36)**
>279.26	0.20 (0.16–0.25)**	0.17 (0.14–0.22)**	0.12 (0.08–0.16)**
Cereal fiber intake (mg/kg/day)			
≤ 47.65	1.00 (ref.)	1.00 (ref.)	1.00 (ref.)
>47.65–79.82	0.79 (0.63–0.96)*	0.75 (0.61–0.93)**	0.68 (0.54–0.87)**
>79.82–126.75	0.64 (0.52–0.78)**	0.61 (0.50–0.74)**	0.57 (0.45–0.72)**
>126.75	0.32 (0.26–0.40)**	0.31 (0.26–0.39)**	0.25 (0.19–0.33)**
Fruit fiber intake (mg/kg/day)			
≤ 0.66	1.00 (ref.)	1.00 (ref.)	1.00 (ref.)
>0.66–20.07	1.09 (0.90–1.33)	1.00 (0.82–1.21)	0.98 (0.79–1.23)
>20.07–48.56	0.79 (0.64–0.96)*	0.69 (0.57–0.84)**	0.75 (0.60–0.94)*
>48.56	0.44 (0.36–0.53)**	0.37 (0.30–0.45)**	0.41 (0.33–0.52)**
Vegetable fiber intake (mg/kg/day)			
≤ 14.71	1.00 (ref.)	1.00 (ref.)	1.00 (ref.)
>14.71–32.36	1.10 (0.90–1.34)	1.07 (0.88–1.31)	1.11 (0.89–1.38)
>32.36–57.62	0.68 (0.54–0.85)**	0.62 (0.49–0.79)**	0.65 (0.49–0.85)**
>57.62	0.41 (0.33–0.51)**	0.37 (0.29–0.47)**	0.42 (0.32–0.56)**

OR, odds ratio; CI, confidence interval. Model 1 adjusted for age and sex. Model 2 adjusted for age, sex, race, education level, somking status, hypertension, diabetes, physical activity, income level, daily average energy intake, UA and TC. The quartile of dietary fiber intake was used as the reference group. Results are survey-weighted. *p < 0.05;**p < 0.01.

Associations of dietary fiber intake with NAFLD in stratified analyses by age and sex are presented in Supplementary Table 2 and Supplementary Table 3, respectively. The inverse associations of total fiber and cereal, fruit, and vegetable fiber intakes with NAFLD were observed in all models, irrespective of sex. In model 2, the ORs (95% CI) of NAFLD in male participants for the highest vs. lowest quartile were 0.15 (0.10–0.24) for total fiber intake, 0.31 (0.22–0.43) for cereal fiber intake, 0.46 (0.34–0.61) for fruit fiber intake, and 0.52 (0.34–0.80) for vegetable fiber intake. The corresponding values in female participants were 0.08 (0.05–0.13), 0.20 (0.12–0.32), 0.36 (0.26–0.48), and 0.34 (0.23–0.49), respectively. Multivariate analysis (model 2) indicated that for participants aged <45 years, the ORs of the NAFLD group for quartile 4 of total fiber, cereal fiber, fruit fiber, and vegetable fiber intakes, compared with quartile 1, were 0.14 (0.08–0.24), 0.29 (0.19–0.46), 0.49 (0.33–0.72), and 0.44 (0.28–0.68), respectively. For participants aged more than 45 years, the corresponding values were 0.10 (0.07–0.15), 0.22 (0.16–0.31), 0.34 (0.25–0.47), and 0.41 (0.28–0.59), respectively.

**Figure 2:**
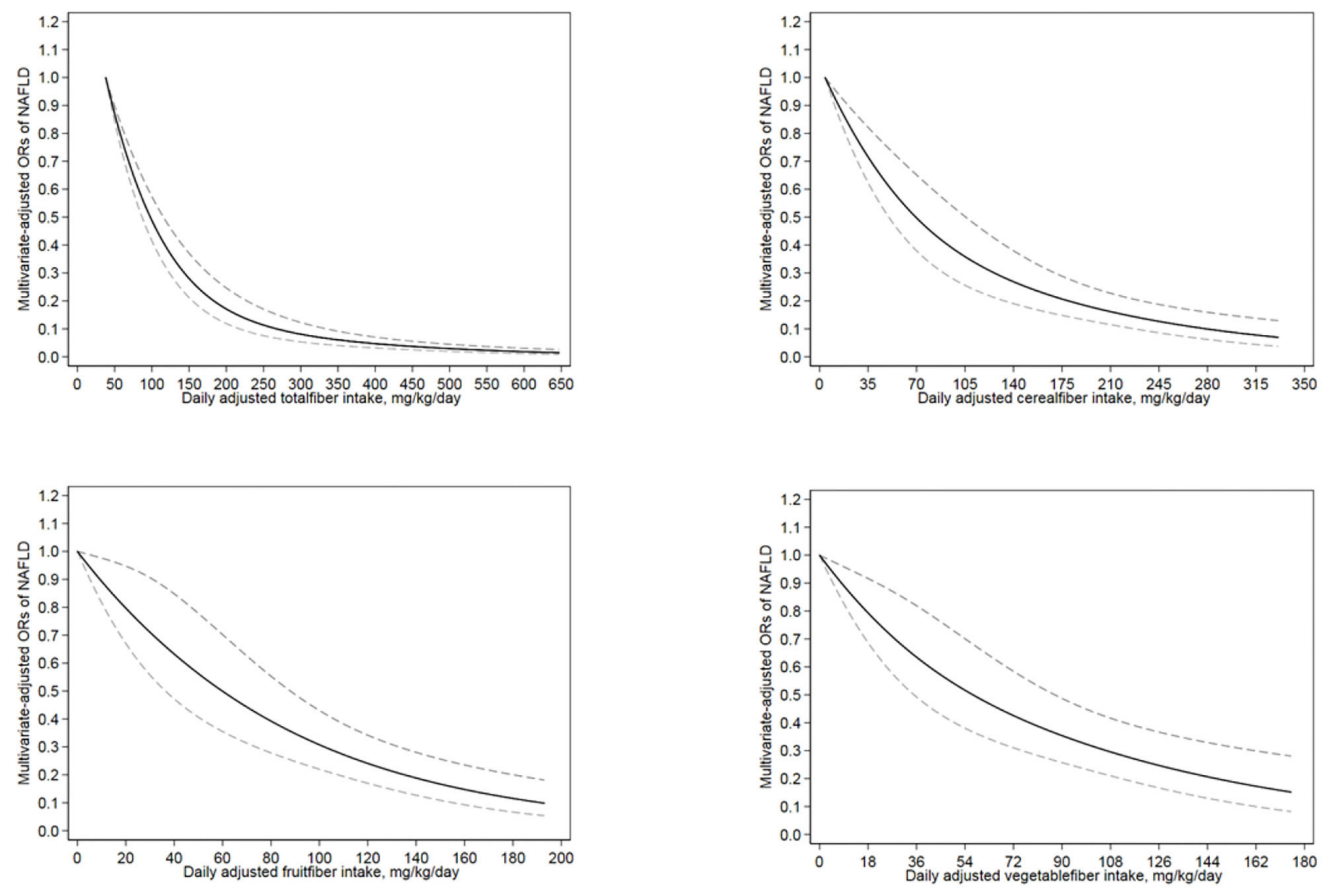
The restricted cubic spline model showed a dose-response relationship between total, cereal, fruit and vegetable dietary fiber intake per kilogram of body weight per day and NAFLD. The lowest level of total fiber intake (38 mg/kg/day), cereal fiber intake (4 mg/kg/day), fruit fiber intake (0 mg/kg/day) and vegetable fiber intake (0 mg/kg/day) were used as the reference group, respectively. Adjustments were made according to age, sex, race, education level, smoking status, income level, hypertension, diabetes, vigorous recreational activity, average energy intake, UA and TC. The solid line and the dotted line represent the estimated OR and the corresponding 95%CI, respectively. OR, odds ratio.

Figure 2 illustrate the associations of total fiber and cereal, fruit, and vegetable fiber intakes with NAFLD in the restricted cubic spline model. The correlation between total fiber intake and NAFLD was non-linearly negative (for non-linearity, *p* < 0.01). With an increase in total fiber intake, the risk of NAFLD decreased and reached a plateau at approximately 293 mg/kg/day (OR = 0.08; 95% CI = 0.05–0.12). Cereal fiber, fruit fiber, and vegetable fiber intakes exhibited a linear inverse association with NAFLD risk (for non-linearity, *p* = 0.32, 0.90, and 0.65, respectively). In addition, when the intake reached 5 mg/kg/day (OR = 0.99; 95% CI = 0.98–0.99) for cereal fiber, 3 mg/kg/day (OR = 0.97; 95% CI = 0.94–0.99) for fruit fiber, and 2 mg/kg/day (OR = 0.97; 95% CI = 0.96–0.99) for vegetable fiber, all these sources of dietary fiber exhibited significant protective effects against NAFLD.

## Discussion

This nationally representative cross-sectional study demonstrated an inverse correlation between total dietary fiber intake (mg/kg/d) and NAFLD risk in the general adult population in the United States after adjusting for multiple potential confounding factors. In the analysis stratified by age (<45 y and ≥45 y groups) and sex (model 2), the inverse association between total fiber intake and NAFLD was still statistically significant. We further studied the relationship between dietary fiber intake from different sources and NAFLD risk. Our results indicated that dietary intakes of cereal, fruit, and vegetable fiber were negatively correlated with NAFLD risk.

We also found a non-linear relationship between total fiber intake and NAFLD risk; an increase in total fiber intake from 38 mg/kg/d to 117 mg/kg/d (Figure 2) decreased the risk of NAFLD by 60%. In addition, cereal, fruit, and vegetable fiber intakes exhibited linear inverse associations with NAFLD risk. To our knowledge, this is the first population-based study to explore the dose-response relationship between dietary intakes of fiber from different sources and NAFLD.

To date, several studies have examined the associations between dietary fiber intake and NAFLD. A cross-sectional study on Dutch adults (general population) demonstrated low consumption of dietary fiber among participants with high fatty liver index (26). Another study conducted in China demonstrated a negative association between total dietary fiber intake and NAFLD (27). A case–control study in Iran also showed lower dietary fiber intake in patients with NAFLD than among healthy controls (28). Thus, our finding indicating an inverse association between total dietary fiber intake and NAFLD is consistent with the findings of the aforementioned studies. However, a cross-sectional study in Israel found no significant difference in dietary fiber intake between NAFLD and control groups (29). Moreover, one cross-sectional study in China revealed higher dietary fiber intake in the NAFLD group than in the control group (30). Notably, neither of these two studies analyzed the relationship between dietary fiber intake and NAFLD risk after adjusting for confounding factors.

Although the biological mechanisms underlying the association between dietary fiber intake and NAFLD are poorly understood, some possible mechanisms for the negative correlation between dietary fiber intake and NAFLD have been proposed; specifically, key roles of insulin resistance, hepatic lipid metabolism, and intestinal floral changes in the pathophysiological process of NAFLD have been identified (39–42). Dietary fiber intake may delay gastric emptying and decrease postprandial blood glucose (39). In addition, studies have shown that dietary fiber may promote lipid excretion (40). Moreover, dietary fiber is fermented by intestinal microorganisms to produce short-chain fatty acids (propionic acid, butyric acid, etc.), which improve insulin sensitivity, and regulate hepatic lipid metabolism (41, 42).

This study has several strengths. First, we assessed the dose-response relationship between different sources of dietary fiber intake and NAFLD risk for the first time. Second, we used data from a large nationally representative survey, which increased the statistical power and reliability of the results. Third, we established a negative correlation between dietary fiber intake and NAFLD that was statistically significant even after adjusting for potential confounding factors.

Nevertheless, our research also has some limitations. First, our study was cross-sectional in design and could not determine the causal relationship between dietary fiber intake and NAFLD risk. Second, dietary data were calculated on the basis of two 24-h recall interviews that may have caused recall bias. Third, although the USFLI used to define NAFLD possessed a superior sensitivity (31), USFLI is unable to stage NAFLD, and the relationship between dietary fiber and NAFLD severity is unclear. Moreover, it should be emphasized that NAFLD in this study, was not clinically diagnosed and was merely estimated from indices. Further well-designed researches are needed in the future.

## Conclusions

In conclusion, dietary intakes of total fiber and cereal, fruit, and vegetable fiber are negatively associated with NAFLD risk in the general adult population in the United States. General U.S. adults should be recommended to increase dietary fiber intake to prevent NAFLD. In addition, large prospective studies are needed to validate our findings.

## Data Availability Statement

The datasets generated for this study can be found in online repositories. The names of the repository/repositories and accession number(s) can be found below: https://www.cdc.gov/nchs/nhanes/.

## Ethics Statement

Written informed consent was obtained from the individual(s) for the publication of any potentially identifiable images or data included in this article.

## Author Contributions

YS and AY designed the study. LM and YQ acquired the data. HZ and JC analyzed the data. HZ drafted the manuscript and YS critically revised the manuscript. All authors read and approved the final manuscript.

## Conflict of Interest

The authors declare that the research was conducted in the absence of any commercial or financial relationships that could be construed as a potential conflict of interest.
