# Supplementary material for: Association Between Dietary Fiber Intake and Non-alcoholic Fatty Liver Disease in Adults
Source: Front Nutr. 2020 Nov 19;7:593735. doi: 10.3389/fnut.2020.593735 (PMC7710900; doi:10.3389/fnut.2020.593735)
Supplement: Supplementary file 1 [file Table_3.DOCX]

| **Supplementary Table1** Weighted ORs and 95% CIs for NAFLD according to the quartiles of dietary fiber intake(mg/kcal/day) | | | |
| --- | --- | --- | --- |
|  | Crude | Model1 | Model2 |
|  | OR(95%CI) | OR(95%CI) | OR(95%CI) |
| Total fiber intake(mg/kcal/day) |  |  |  |
| ≤6.91 | 1.00(ref.) | 1.00(ref.) | 1.00(ref.) |
| >6.91-9.99 | 0.83(0.69-1.01) | 0.78(0.64-0.94)* | 0.87(0.67-1.14) |
| >9.99 | 0.72(0.62-0.85)** | 0.64(0.54-0.76)** | 0.74(0.58-0.93)* |
| Cereal fiber intake(mg/kcal/day) |  |  |  |
| ≤2.67 | 1.00(ref.) | 1.00(ref.) | 1.00(ref.) |
| >2.67-4.46 | 1.03(0.89-1.18) | 1.02(0.89-1.18) | 1.05(0.85-1.29) |
| >4.46 | 0.87(0.75-1.00) | 0.85(0.74-0.99)* | 0.91(0.73-1.13) |
| Fruit fiber intake(mg/kcal/day) |  |  |  |
| ≤0.27 | 1.00(ref.) | 1.00(ref.) | 1.00(ref.) |
| >0.27-1.58 | 0.87(0.73-1.04) | 0.78(0.65-0.93)** | 0.89(0.72-1.10) |
| >1.58 | 0.64(0.54-0.76)** | 0.56(0.46-0.67)** | 0.73(0.57-0.92)** |
| Vegetable fiber intake(mg/kcal/day) |  |  |  |
| ≤0.89 | 1.00(ref.) | 1.00(ref.) | 1.00(ref.) |
| >0.89-2.02 | 1.00(0.87-1.15) | 0.99(0.85-1.14) | 1.03(0.84-1.26) |
| >2.02 | 0.83(0.70-0.98)* | 0.76(0.63-0.92)** | 0.80(0.61-1.04) |
| OR, odds ratio; CI, confidence interval. Model 1 adjusted for age and gender. Model 2 adjusted for age, gender, race, somking status, hypertension, diabetes, physical activity, BMI, UA and TC. The quartile of dietary fiber intake was used as the reference group. Results are survey-weighted.**p<0.05;**p<0.01.* | | | |

| **Supplementary Table2** Weighted ORs and 95% CIs for NAFLD according to the quartiles of dietary fiber intake, stratified by gender | | | |
| --- | --- | --- | --- |
|  | Crude | Model1 | Model2 |
|  | OR(95%CI) | OR(95%CI) | OR(95%CI) |
| Male |  |  |  |
| Total fiber intake(mg/kg/day) |  |  |  |
| ≤130.89 | 1.00(ref.) | 1.00(ref.) | 1.00(ref.) |
| >130.89-195.50 | 0.69(0.54-0.89)** | 0.68(0.53-0.86)** | 0.66(0.48-0.92)* |
| >195.50-281.93 | 0.42(0.32-0.56)** | 0.39(0.29-0.53)** | 0.31(0.21-0.45)** |
| >281.93 | 0.23(0.17-0.33)** | 0.22(0.16-0.31)** | 0.15(0.10-0.24)** |
| Cereal fiber intake(mg/kg/day) |  |  |  |
| ≤50.52 | 1.00(ref.) | 1.00(ref.) | 1.00(ref.) |
| >50.52-86.19 | 0.93(0.72-1.19)** | 0.89(0.69-1.15) | 0.92(0.67-1.26) |
| >86.19-135.17 | 0.64(0.49-0.84)** | 0.63(0.48-0.83)** | 0.61(0.44-0.86)** |
| >128.91 | 0.34(0.25-0.46)** | 0.35(0.26-0.47)** | 0.31(0.22-0.43)** |
| Fruit fiber intake(mg/kg/day) |  |  |  |
| ≤0 | 1.00(ref.) | 1.00(ref.) | 1.00(ref.) |
| >0-15.54 | 0.93(0.71-1.20) | 0.84(0.64-1.11) | 0.91(0.66-1.26) |
| >15.54-42.36 | 0.80(0.61-1.05) | 0.68(0.52-0.90)** | 0.74(0.54-1.00) |
| >42.36 | 0.49(0.38-0.62)** | 0.41(0.31-0.53)** | 0.46(0.34-0.61)** |
| Vegetable fiber intake(mg/kg/day) |  |  |  |
| ≤14.93 | 1.00(ref.) | 1.00(ref.) | 1.00(ref.) |
| >14.93-31.89 | 1.09(0.82-1.43) | 1.05(0.80-1.38) | 1.06(0.77-1.47) |
| >31.89-56.57 | 0.78(0.59-1.03) | 0.72(0.55-0.96)* | 0.80(0.60-1.08) |
| >56.57 | 0.50(0.35-0.72)** | 0.44(0.30-0.65)** | 0.52(0.34-0.80)** |
| Female |  |  |  |
| Total fiber intake(mg/kg/day) |  |  |  |
| ≤130.97 | 1.00(ref.) | 1.00(ref.) | 1.00(ref.) |
| >130.97-189.53 | 0.75(0.56-1.00) | 0.67(0.51-0.90)** | 0.52(0.35-0.77)** |
| >189.53-274.61 | 0.35(0.27-0.47)** | 0.32(0.24-0.42)** | 0.22(0.15-0.32)** |
| >274.61 | 0.15(0.11-0.22)** | 0.14(0.10-0.19)** | 0.08(0.05-0.13)** |
| Cereal fiber intake(mg/kg/day) |  |  |  |
| ≤47.65 | 1.00(ref.) | 1.00(ref.) | 1.00(ref.) |
| >47.65-77.62 | 0.69(0.52-0.90)** | 0.66(0.50-0.87)** | 0.52(0.38-0.69)** |
| >77.62-119.69 | 0.58(0.44-0.77)** | 0.56(0.42-0.74)* | 0.50(0.35-0.72)** |
| >119.69 | 0.28(0.20-0.40)** | 0.29(0.20-0.41)** | 0.20(0.12-0.32)** |
| Fruit fiber intake(mg/kg/day) |  |  |  |
| ≤1.79 | 1.00(ref.) | 1.00(ref.) | 1.00(ref.) |
| >1.79-21.96 | 1.29(0.96-1.73) | 1.15(0.86-1.54) | 1.04(0.73-1.49) |
| >21.96-50.05 | 0.94(0.70-1.26) | 0.80(0.60-1.06) | 0.84(0.60-1.17) |
| >50.05 | 0.42(0.33-0.54)** | 0.34(0.26-0.43)** | 0.36(0.26-0.48)** |
| Vegetable fiber intake(mg/kg/day) |  |  |  |
| ≤17.46 | 1.00(ref.) | 1.00(ref.) | 1.00(ref.) |
| >17.46-35.88 | 1.06(0.85-1.32) | 0.98(0.78-1.24) | 1.05(0.79-1.38) |
| >35.88-62.80 | 0.62(0.49-0.78)** | 0.56(0.44-0.71)** | 0.59(0.43-0.79)** |
| >62.80 | 0.35(0.26-0.46)** | 0.30(0.23-0.39)** | 0.34(0.23-0.49)** |
| OR, odds ratio; CI, confidence interval. Model 1 adjusted for age and gender. Model 2 adjusted for age, gender, race, education level, somking status, hypertension, diabetes, physical activity, income level, daily average energy intake, UA and TC. The quartile of dietary fiber intake was used as the reference group. Results are survey-weighted.**p<0.05;**p<0.0*1. | | | |

| **Supplementary Table3** Weighted ORs and 95% CIs for NAFLD according to the quartiles of dietary fiber intake, stratified by age | | | |
| --- | --- | --- | --- |
|  | Crude | Model1 | Model2 |
|  | OR(95%CI) | OR(95%CI) | OR(95%CI) |
| <45 years |  |  |  |
| Total fiber intake(mg/kg/day) |  |  |  |
| ≤128.70 | 1.00(ref.) | 1.00(ref.) | 1.00(ref.) |
| >128.70-191.02 | 0.66(0.49-0.89)** | 0.65(0.48-0.88)** | 0.58(0.38-0.90)** |
| >191.02-279.26 | 0.36(0.26-0.50)** | 0.35(0.25-0.49)** | 0.26(0.16-0.43)** |
| >279.26 | 0.25(0.18-0.36)** | 0.24(0.17-0.34)** | 0.14(0.08-0.24)** |
| Cereal fiber intake(mg/kg/day) |  |  |  |
| ≤47.65 | 1.00(ref.) | 1.00(ref.) | 1.00(ref.) |
| >47.65-79.82 | 0.80(0.60-1.08) | 0.80(0.60-1.06) | 0.79(0.55-1.14) |
| >79.82-126.75 | 0.73(0.55-0.97)* | 0.71(0.54-0.93)* | 0.66(0.44-0.98)* |
| >126.75 | 0.39(0.28-0.55)** | 0.37(0.26-0.51)** | 0.29(0.19-0.46)** |
| Fruit fiber intake(mg/kg/day) |  |  |  |
| ≤0.66 | 1.00(ref.) | 1.00(ref.) | 1.00(ref.) |
| >0.66-20.07 | 1.04(0.77-1.42) | 1.06(0.78-1.44) | 1.04(0.71-1.53) |
| >20.07-48.56 | 0.72(0.53-0.98)* | 0.75(0.56-1.00) | 0.90(0.64-1.28) |
| >48.56 | 0.42(0.31-0.58)** | 0.43(0.31-0.59)** | 0.49(0.33-0.72)** |
| Vegetable fiber intake(mg/kg/day) |  |  |  |
| ≤14.71 | 1.00(ref.) | 1.00(ref.) | 1.00(ref.) |
| >14.71-32.36 | 1.05(0.79-1.40) | 1.05(0.79-1.41) | 0.98(0.69-1.40) |
| >32.36-57.62 | 0.59(0.44-0.81)** | 0.60(0.45-0.80)** | 0.59(0.51-0.86)** |
| >57.62 | 0.37(0.26-0.52)** | 0.38(0.26-0.54)** | 0.44(0.28-0.68)** |
| ≥45 years |  |  |  |
| Total fiber intake(mg/kg/day) |  |  |  |
| ≤128.70 | 1.00(ref.) | 1.00(ref.) | 1.00(ref.) |
| >128.70-191.02 | 0.69(0.53-0.90)** | 0.71(0.54-0.92)* | 0.61(0.44-0.85)** |
| >191.02-279.26 | 0.39(0.30-0.51)** | 0.38(0.29-0.50)** | 0.28(0.21-0.38)** |
| >279.26 | 0.15(0.11-0.20)** | 0.15(0.11-0.19)** | 0.10(0.07-0.15)** |
| Cereal fiber intake(mg/kg/day) |  |  |  |
| ≤47.65 | 1.00(ref.) | 1.00(ref.) | 1.00(ref.) |
| >47.65-79.82 | 0.72(0.55-0.94)* | 0.72(0.56-0.94)* | 0.64(0.46-0.89)** |
| >79.82-126.75 | 0.56(0.43-0.72)** | 0.55(0.42-0.72)** | 0.50(0.37-0.67)** |
| >126.75 | 0.30(0.23-0.39)** | 0.28(0.22-0.37)** | 0.22(0.16-0.31)** |
| Fruit fiber intake(mg/kg/day) |  |  |  |
| ≤0.66 | 1.00(ref.) | 1.00(ref.) | 1.00(ref.) |
| >0.66-20.07 | 0.94(0.73-1.21) | 0.96(0.75-1.24) | 0.85(0.62-1.17) |
| >20.07-48.56 | 0.64(0.51-0.81)** | 0.67(0.53-0.84)** | 0.63(0.49-0.81)** |
| >48.56 | 0.33(0.25-0.44)** | 0.35(0.26-0.46)** | 0.34(0.25-0.47)** |
| Vegetable fiber intake(mg/kg/day) |  |  |  |
| ≤14.71 | 1.00(ref.) | 1.00(ref.) | 1.00(ref.) |
| >14.71-32.36 | 1.07(0.81-1.41) | 1.09(0.82-1.43) | 1.21(0.91-1.61) |
| >32.36-57.62 | 0.63(0.46-0.87)** | 0.64(0.47-0.88)** | 0.67(0.48-0.94)* |
| >57.62 | 0.37(0.27-0.50)** | 0.38(0.27-0.51)** | 0.41(0.28-0.59)** |
| OR, odds ratio; CI, confidence interval. Model 1 adjusted for age and gender. Model 2 adjusted for age, gender, race, education level, somking status, hypertension, diabetes, physical activity, income level, daily average energy intake, UA and TC. The quartile of dietary fiber intake was used as the reference group. Results are survey-weighted.**p<0.05;**p<0.0*1. | | | |
